# Optimizing protein delivery rate from silk fibroin hydrogel using silk fibroin-mimetic peptides conjugation

**DOI:** 10.1038/s41598-024-53689-7

**Published:** 2024-02-23

**Authors:** Jaturong Promsuk, Juthatip Manissorn, Chavee Laomeephol, Jittima Amie Luckanagul, Apipon Methachittipan, Khaow Tonsomboon, Ratchapol Jenjob, Su-Geun Yang, Peerapat Thongnuek, Kittikhun Wangkanont

**Affiliations:** 1 Department of Biochemistry, Center of Excellence for Molecular Biology and Genomics of Shrimp, Faculty of Science, Chulalongkorn University, Bangkok, 10330 Thailand; 2 Department of Biochemistry, Center of Excellence in Molecular Crop, Faculty of Science, Chulalongkorn University, Bangkok, 10330 Thailand; 3 Biomedical Materials and Devices for Revolutionary Integrative Systems Engineering Research Unit (BMD-RISE), Faculty of Engineering, Chulalongkorn University, Bangkok, 10330 Thailand; 4 Department of Pharmaceutics and Industrial Pharmacy, Faculty of Pharmaceutical Sciences, Chulalongkorn University, Bangkok, 10330 Thailand; 5 Nano Engineering Program, International School of Engineering, Faculty of Engineering, Chulalongkorn University, Bangkok, 10330 Thailand; 6 National Center for Genetic Engineering and Biotechnology (BIOTEC), National Science and Technology Development Agency (NSTDA), Khlong Luang, 12120 Pathum Thani Thailand; 7 Department of Biomedical Science, BK21 FOUR Program in Biomedical Science and Engineering, Inha University College of Medicine, Incheon, 22332 Republic of Korea; 8 Biomedical Engineering Program, Faculty of Engineering, Chulalongkorn University, Bangkok, 10330 Thailand; 9 Biomedical Engineering Research Center, Faculty of Engineering, Chulalongkorn University, Bangkok, 10330 Thailand

**Keywords:** Biomaterials, Soft materials, Bioconjugate chemistry, Biopolymers, Mechanical properties, Polymer characterization, Biomedical engineering, Biomaterials, Biomimetics, Protein delivery

## Abstract

Controlled release of proteins, such as growth factors, from biocompatible silk fibroin (SF) hydrogel is valuable for its use in tissue engineering, drug delivery, and other biological systems. To achieve this, we introduced silk fibroin-mimetic peptides (SFMPs) with the repeating unit (GAGAGS)_n_. Using green fluorescent protein (GFP) as a model protein, our results showed that SFMPs did not affect the GFP function when conjugated to it. The SFMP-GFP conjugates incorporated into SF hydrogel did not change the gelation time and allowed for controlled release of the GFP. By varying the length of SFMPs, we were able to modulate the release rate, with longer SFMPs resulting in a slower release, both in water at room temperature and PBS at 37 °C. Furthermore, the SF hydrogel with the SFMPs showed greater strength and stiffness. The increased β-sheet fraction of the SF hydrogel, as revealed by FTIR analysis, explained the gel properties and protein release behavior. Our results suggest that the SFMPs effectively control protein release from SF hydrogel, with the potential to enhance its mechanical stability. The ability to modulate release rates by varying the SFMP length will benefit personalized and controlled protein delivery in various systems.

## Introduction

Sustained release in drug delivery systems is crucial for ensuring effective drug levels^1^. Silk fibroin (SF), a natural biopolymer derived from domestic silkworms (*Bombyx mori*), is a promising candidate for drug delivery-system development due to its biocompatibility, mechanical strength, and resistance to mammalian proteases^2–4^. The silk protein is composed of two main components: fibroin, the main structural protein, and sericin, which acts as a supportive glue^5,6^. The primary structure of SF features repetitive amino acid sequences, with the predominant sequence being the glycine-alanine-glycine-alanine-glycine-serine (GAGAGS) repeat^7^. The GAGAGS repeat forms crystalline structures during sol–gel transition by adopting highly ordered secondary structures, such as β-sheet^8^.

Various forms of SF-based materials have been developed as drug carriers. They were loaded with small-molecule drugs or bioactive proteins. Simple loading of the small molecules in the SF hydrogel often showed the undesirable burst release, resulting in depletion of the bioactive molecules in a short time. For example, SF hydrogel burst-released diclofenac sodium, and reached the equilibrium concentration within 5–10 h^9^. It was suggested that hindering drug diffusion from the SF hydrogel could prevent the burst release of a water-soluble drug^10^. Bioactive proteins, which are larger than small-molecule drugs, loaded into SF materials exhibited 3 phases of the release before reaching the equilibrium: the burst-release phase, a short lag phase, and a continuous phase^11,12^. Using horseradish peroxidase and lysozyme as model protein drugs, it was hypothesized that the burst release was caused by the initial diffusion of the soluble bioactive protein incorporated in the amorphous regions of the SF. The burst release was overcome by increasing the crystallinity of the SF. The crystalline β-sheets acted as a diffusion barrier that trapped the protein, thus slowed down the release^11,12^. The lag and continuous phase resulted from the degradation of the crystalline regions that untrapped the bioactive protein from the SF matrix^11,12^. This highlights the importance of overcoming the burst release in order to utilize SF hydrogel in controlled release of bioactive proteins in tissue engineering applications and other application in various biological systems.

One strategy to hinder the initial burst-release phase is to append peptides mimicking the structural domains of the hydrogel. Short collagen-mimicking peptides that replaced the proline and hydroxyproline in the Gly-X-Y domains with fluoroproline were well-retained in the collagen hydrogel, presumably by interacting with the native Gly-X-Y domain^13^. The biomimetic peptide could be used for the incorporation of bioactive compounds into hydrogels.

**Figure 1:**
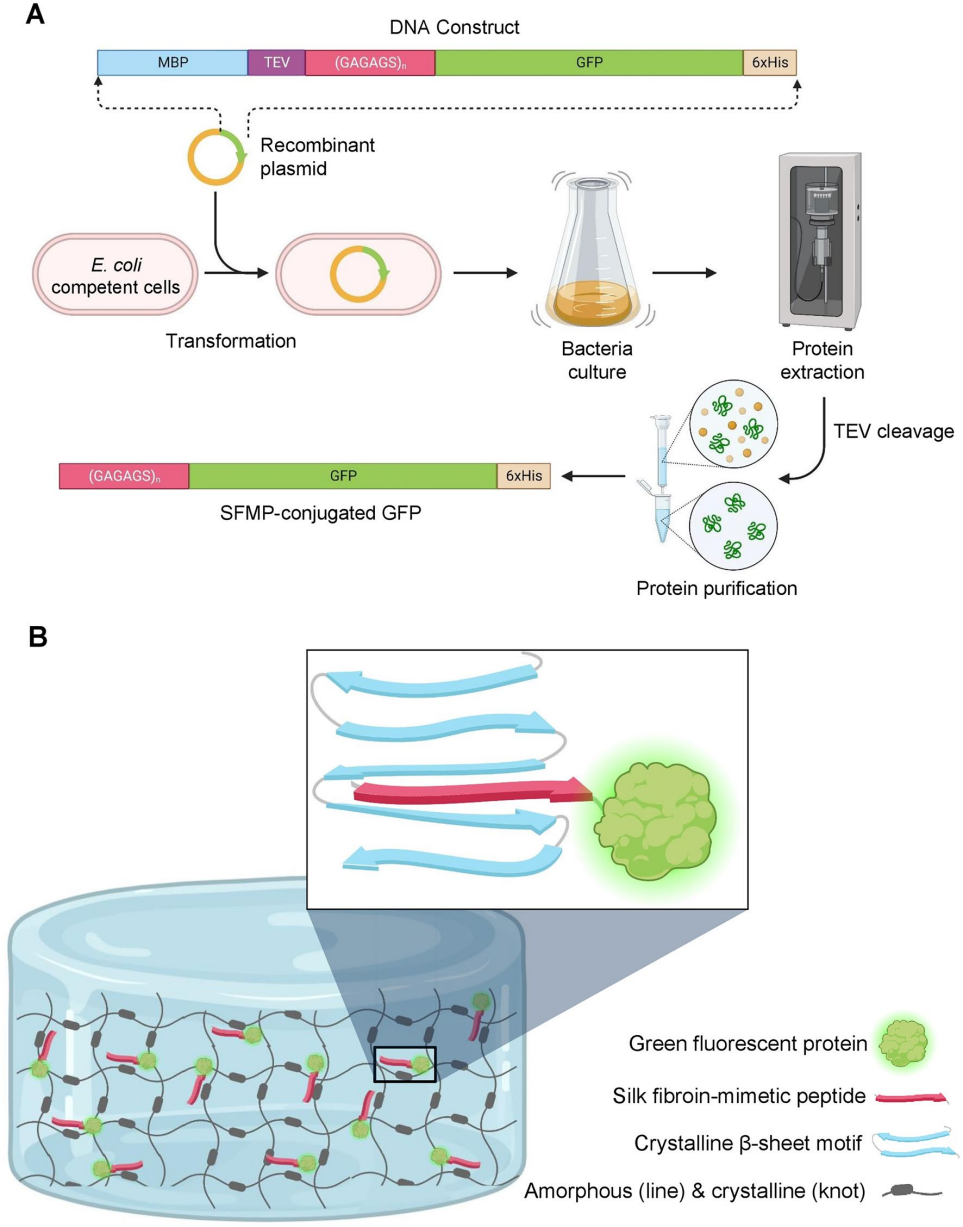
Schematics of the SFMPs. (**A**) The production of SFMP-conjugated protein used in this study ((GAGAGS)_n_-GFP). (**B**) The design concept illustrating the possible interaction between the SFMPs and the crystalline domains that should hinder the initial burst release by diffusion and result in sustained release of the GFP.

In this work, we created recombinant peptides mimicking the β-sheet forming domain of the SF. The SF-mimetic peptides (SFMPs) containing varied numbers of GAGAGS repeats, (GAGAGS)_n_, were conjugated to the green fluorescent protein (GFP) by genetic engineering (Fig. 1A). The GFP was used as a model protein to demonstrate our design concept because the release kinetics could be readily monitored. We hypothesized that the GAGAGS should interact with the analogous β-sheet structures in the hydrogel, hindering the burst release of the protein model (Fig. 1B). Moreover, because the GAGAGS repeating sequence is identical to those in silk fibroin, SFMPs should also be a biocompatible tag. Therefore, we analyzed the release profiles of the (GAGAGS)_n_-GFP and examined whether the SFMPs influenced the hydrogel structure.

## Materials and methods

### Construction of the SFMP-conjugated GFP expression plasmids

The expression construct for (GAGAGS)_3_-GFP was initially constructed by amplifying GFP from pEGFP-N1 (Clontech; San Jose, CA) by two forward primers (primer A and primer B) and primer C as the reverse primer (Table S1). The PCR product was cloned into the NcoI and NotI sites of pET28a (Novagen; Medison, WI). This plasmid was designated as pET28a-(GAGAGS)_3_-GFP. However, upon induction of gene expression in *Escherichia coli* Tuner (DE3), no recombinant protein expression was observed. Thus, GAGAGS-conjugated GFPs were expressed with the maltose-binding protein (MBP) tag at the N termini followed by the tobacco etch virus protease (TEV) cleavage to allow cleavage of the GAGAGS-tagged GFPs. These expression plasmids were constructed by cloning the TEV-(GAGAGS)_n_-GFP PCR fragments into the NcoI and BamHI sites of pMAL-c5x (New England Biolabs; Ipswich, MA). For the construction of the pMALc5x-TEV-(GAGAGS)_3_-GFP, primer D and F were used to amplify the pET28a-(GAGAGS)_3_-GFP plasmid. Primer E and primer F were used to generate the pMAL-c5x-TEV-GFP plasmid using pET28a-(GAGAGS)_3_-GFP as the template. For pMAL-c5x-TEV-GAGAGS-GFP, primer G and primer F were used with pMAL-c5x-TEV-GFP as the template. For pMALc5x-TEV-(GAGAGS)_2_-GFP, primer H and primer F were used with pMAL-c5x-TEV-GAGAGS-GFP as the template. Primers I and F were used to amplify the pMALc5x-TEV-(GAGAGS)_3_-GFP template to obtain a PCR product. The PCR product was then used as a template for amplification with primer J and primer F. pMALc5x-TEV-(GAGAGS)_5_-GFP could be isolated upon cloning of the PCR product, as well as pMALc5x-TEV-(GAGAGS)_4_-GFP which likely arose as a mispriming product. For pMALc5x-TEV-(GAGAGS)_6_-GFP, the PCR product of primers I and F was also used as a template for amplification with primers K and primer F prior to cloning. The nucleotide sequences of all expression plasmids were confirmed by DNA sequencing.

### (GAGAGS)_n_-GFP expression

*Escherichia coli* Tuner (DE3) that had been transformed with the pMALc5x-TEV-(GAGAGS)_n_-GFP plasmid construct were grown in 100 mL Terrific Broth with 100 µg/mL ampicillin for 16–18 h at 37 °C as a starter culture. The starter culture was inoculated into 500 mL Terrific Broth at 3% concentration. The culture was grown at 37 °C until the OD_600_ value reached 0.4–0.6. IPTG was then added to 0.1 mM, and protein expression was allowed to proceed for 6 h at 30 °C. Cells were pelleted by centrifugation for 10 min at 6000×*g* at 4 °C and stored at − 80 °C until protein purification.

### (GAGAGS)_n_-GFP purification

To purify (GAGAGS)_n_-GFP protein, cell pellets were re-suspended in 30 mL of buffer A (25 mM HEPES, 150 mM NaCl and 25 mM imidazole, pH 7.5) containing 0.1 mg/mL lysozyme and incubated for 30 min at 4 °C. Thereafter, phenylmethylsulfonyl fluoride (PMSF) was added to 1 mM. The cells were disrupted using sonication and followed by centrifugation (at 40,000×*g*, 4 °C, 30 min). The supernatant was loaded onto a 3-mL Ni–NTA agarose (GoldBio; St. Louis, MO) column pre-equilibrated with buffer A. The column was washed with buffer A, and the bound protein was eluted with buffer A containing 250 mM imidazole. The obtained MBP-TEV-(GAGAS)_n_-GFP protein was digested with TEV protease [Ref: PMID: 11809930] for 48 h at 4 °C. The cleaved protein was dialyzed against buffer A then re-applied to the 3-mL Ni–NTA agarose column. The (GAGAGS)_n_-GFP protein was eluted with buffer A containing 250 mM imidazole and dialyzed against buffer B (20 mM Tris, pH 7.0). The protein was subsequently loaded onto a 1-mL Q-Sepharose column (GE Healthcare; Uppsala, Sweden) and pre-equilibrated in buffer B. The column was washed with a step gradient of buffer B that was supplemented with 60, 70, 80, and 90 mM NaCl for removal of the TEV protease. The (GAGAGS)_n_-GFP protein was eluted with buffer B that had been supplemented with 100 mM NaCl. The purity of the purified protein was examined by SDS-PAGE with Coomassie brilliant blue R staining. The concentration of (GAGAGS)_n_-GFP was measured by the Bradford protein assay (Bio-Rad, Hercules, CA).

### Preparation of the silk fibroin solution

Thai silk cocoons of *Bombyx mori* “Nangnoi Srisaket 1” were manually cleaned to remove visible contaminants. To remove the sericin, the clean silk cocoons (40 g) were boiled for 20 min in 1 L of 0.2 M Na_2_CO_3_ solution. The silk fibers were washed with distilled water until the water appeared colorless and dried for 2 days at room temperature. The dried silk fibers (4 g) were dissolved at 60 °C in 16 mL of 9.3 M LiBr solution. The amber viscous solution of the dissolved SF was obtained after 4 h. To remove the LiBr from the SF solution, the solution was dialyzed for 48 h against deionized water using a dialysis bag with a 12–16 kDa molecular weight cut-off (Sekisui; Osaka, Japan). The dialyzed SF solution was centrifuged to remove the impurities for 20 min at 13,000×*g*, 4 °C.

### Silk fibroin hydrogel fabrication and gelation study

The SF hydrogel was prepared from the SF solution by spontaneous gelation. The SF solution was diluted to 4% (w/v) with 20 mM Tris, pH 7.0. The 4% w/v SF solution was then loaded with 100 µg/mL (GAGAGS)_n_-GFP solutions and incubated at 37 °C until the solution become gelled.

In the gelation study, SF solution was prepared to concentration of 4% w/v and mixed with 100 µg/mL (GAGAGS)_n_-GFP while the control was mixed with 20 mM Tris, pH 7.0 and incubated at 37 °C. The gelation time was determined by measuring the absorbance at 550 nm using microplate reader (BioTek Synergy H1, Agilent Technologies, Inc.; Santa Clara, CA). The gelation time is the time at which the mean optical density reached half-maximum value^14^. The sol–gel transition was recorded every day for 5 days.

### In vitro release study of SFMP-GFP from SF hydrogels

The SF hydrogel containing (GAGAGS)_n_-GFP (10 μg, 5 × 5 × 5 mm) was submerged in 1 mL of deionized water (DI) or PBS, then incubated at room temperature or at 37 °C, respectively. The fluorescence intensity of the GFP (100 μL sample) was measured using the excitation wavelength of 395 nm and the emission wavelength of 510 nm by a microplate reader (BioTek Synergy H1). After each fluorescence measurement, the sample was added back to its respective solution to maintain the total volume of the experiment. The fluorescence intensity was recorded and standardized to the control, which was the GFP at a known concentration. The fluorescence values were presented as relative fluorescence units (RFU), which were later converted to µg using a standard curve. The observation was done every 30 min within the first 2 h and then every 24 h until 7 days.

The initial release during the first 2 h was analysed using linear regression. Non-linear regression analysis was done by fitting the release profiles to the following kinetics models using GraphPad Prism.

The first-order model^15^:

$${\text{log}}\left({q}_{e}-{q}_{t}\right)={\text{log}}{q}_{e}-{k}_{1}t$$

where ${q}_{e}$ is the fluorescence intensity at equilibrium, ${q}_{t}$ is the fluorescence intensity at a specific time point, ${k}_{1}$ is the rate constant for the first-order kinetic model, $t$ is the time point.

The pseudo-second-order model^16^:

$$\frac{t}{{q}_{t}}=\frac{1}{{k}_{2}}{q}_{e}^{2}+\frac{t}{{q}_{e}}$$

where ${q}_{e}$ is the fluorescence intensity at equilibrium, ${q}_{t}$ is the fluorescence intensity at a specific time point, ${k}_{2}$ is the rate constant for the pseudo-second-order kinetic model, $t$ is the time point.

The Korsmeyer-Peppas model^16^:

$$log{ q}_{t}=n{\text{log}}t+{\text{log}}{k}_{KP}$$

where ${q}_{t}$ is the fluorescence intensity at a specific time point, $n$ is the release exponent, $t$ is the time point, ${k}_{KP}$ is the rate constant for the Korsmeyer-Peppas kinetic model.

The Higuchi model^16^:

$${q}_{t}={k}_{H}\sqrt{t}$$

where ${q}_{t}$ is the fluorescence intensity at a specific time point, ${k}_{H}$ is the rate constant for the Higuchi kinetic model, $t$ is the time point.

### Compressive mechanical analysis of SF hydrogels

The SF hydrogel containing 100 µg/mL of (GAGAGS)_n_-GFP were prepared and allowed for gelation. The hydrogels without (GAGAGS)_n_-GFP were the control. Uniaxial mechanical analysis was performed in an unconfined compressive mode using a universal testing machine (Hounsfield H10 KM; Tinius Olsen, UK) equipped with 10 N load cell. The hydrogels were cut into a cylindrical shape with a thickness and a diameter of approximately 8 mm and immersed in 0.01 M phosphate buffer saline (pH 7.4) buffer until performing the tests. The samples were compressed at a displacement speed of 5 mm/min. The tests were conducted in triplicate. The Young’s modulus was calculated from the slope of a stress–strain curve in a linear elastic region, and the yield strength and strain were determined at a maximum value before the hydrogels lost their elastic properties.

### Rheological study of SF hydrogels

Oscillatory mechanical evaluation was performed using a Haake Mars rheometer (Thermo Fisher Scientific) equipped with a 35-mm parallel plate rotor. The hydrogels were gently transferred to the measuring plate with a gap set at 1 mm. The temperature was controlled at 37 °C. A frequency-sweep experiment was conducted with a fixed strain of 0.5%, and the storage modulus (G′) and loss modulus (G″) were collected over a range of frequency from 0.5 to 100 Hz. To identify the shear-thinning of the hydrogels, thixotropic analysis was then performed in a rotational mode with three cycles of 100 s^−1^ and 0.1 s^−1^ shear rate at 37 °C.

### Fourier transform infrared (FTIR) spectroscopy and quantitative analysis of protein secondary structures

SF hydrogels containing 100 µg/mL of (GAGAGS)_n_-GFP were quickly frozen in the liquid nitrogen and lyophilized. The lyophilized SF hydrogels were analyzed by FTIR spectrometer using an attenuated total reflectance (ATR) mode (Nicolet iS50 FTIR, Thermo Scientific Inc.; Waltham, MA). The spectra were collected from 4000 to 400 cm^−1^. Quantitative analysis of protein secondary structures was quantified from the area percentage under defined peaks of Fourier self-deconvoluted FTIR spectra according to an established protocol with slight modifications^8^. Briefly, the amide I spectrum (1725–1575 cm^−1^) was deconvoluted by Omnic 8.0 software (Thermo Scientific) using Voigt line-shape fitting with a half-bandwidth of 25 cm^−1^ and an enhancement factor of 3.0. The Gaussian curve-fitting of the obtained spectrum was then performed using Origin Pro 9.0 software (OriginLab; Northampton, MA). The content of β-sheet structures was determined from the area under peaks located within 1616–1637 cm^−1^ and 1696–1703 cm^−1^. The content of random coil, α-helix, and β-turn was from the peaks in a range of 1638–1655, 1656–1662, and 1663–1696 cm^−1^, respectively^17^.

### X-ray diffraction (XRD) and crystallinity index

The XRD analysis of the freeze-dried samples was performed using an X-ray diffractometer (Rigaku Smartlab 30 kV; Tokyo, Japan) equipped with a fixed monochromator. A Cu Kα radiation source was set to an accelerating voltage of 40 kV with an applied current of 30 mA and a scan speed of 2°/min. The 2θ used was in the range of 5–40°. The diffraction at the 2θ angles of 20.7 and 24.6° is indicative of the SF crystalline fraction of β-sheet structures^18^.

### Biocompatibility test

#### MTT assay

To determine the biocompatibility of (GAGAGS)_n_-GFP, MTT assay was performed on NIH(3T3) fibroblasts. Briefly, the NIH(3T3) cells were cultured in DMEM (Gibco; Carlsbad, CA) supplemented with 10% fetal bovine serum (FBS) and 1% penicillin–streptomycin (HyClone Laboratories Inc., Logan, UT). The culture was maintained in a humidified incubator at 37 °C with 5% CO_2_. The cells were seeded at the density of 1.25 × 10^4^ cells/cm^2^ in 24-well plates for 24 h. The cells were then treated with SF hydrogel containing 10 µg/mL purified (GAGAGS)_n_-GFP in DMEM supplemented with 10% FBS for 48 h. After that, the MTT assay was performed using 3-(4,5-dimethylthiazol-2-yl)-2,5-diphenyltetrazolium bromide. Briefly, the cells were washed with PBS and incubated for 1 h with 0.5 mg/mL MTT solution at 37 °C in the dark. Thereafter, the MTT solution was removed and replaced with dimethyl sulfoxide (DMSO) to extract the precipitated formazan crystals. The solution was subjected to absorbance measurement at 570 nm.

### Statistical analysis

Data were presented as mean ± standard deviation (SD). Statistical analysis was performed using SPSS version 16.0 (SPSS; Chicago, IL). Data were performed using one-way analysis of variance (ANOVA) with Tukey’s post-hoc test. *P*-values less than 0.05 were considered statistically significant.

## Results

### Construction, expression, and purification of (GAGAGS)_n_-GFP proteins

**Figure 2:**
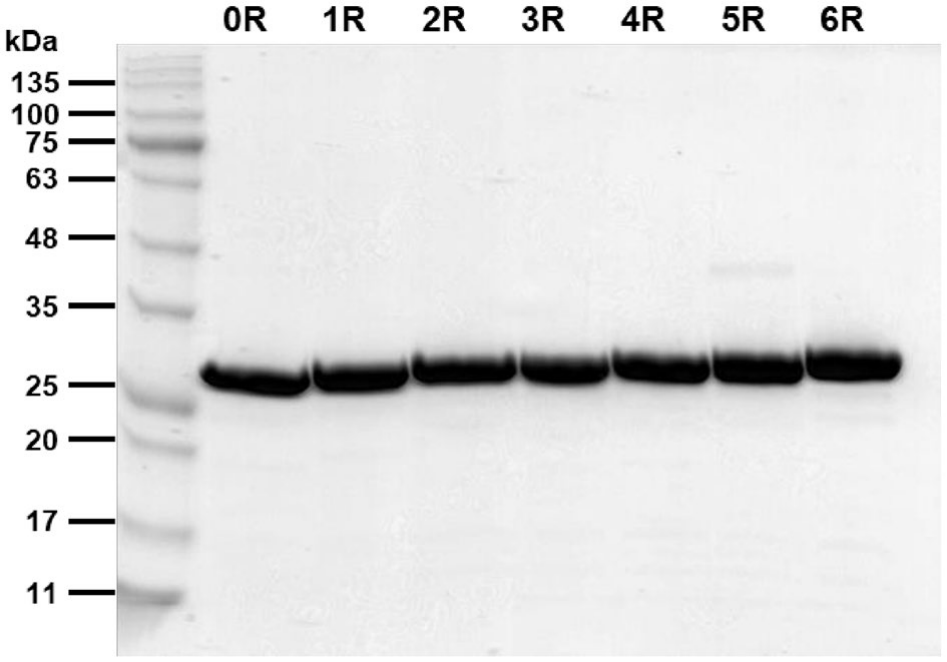
Purity of (GAGAGS)_n_-GFP proteins. SDS-PAGE analysis of the purified (GAGAGS)_n_-GFP where n equals the number of SF repeats. 0R = unconjugated GFP, nR = GFP conjugated to n repeat of GAGAGS. Each lane contained 12 µg of protein.

A DNA sequence coding for the SFMPs with GAGAGS ranging from 0 to 6 repeats ((GAGAGS)_n_, n = 0–6) with a tobacco etch virus (TEV) protease cleavage site at the N terminus was fused to the DNA sequence coding for the green fluorescent protein (GFP) with a C-terminal hexahistidine tag. This construct was inserted downstream of the maltose-binding protein (MBP) gene in the expression vector pMALc5x (Table S2). After plasmid construction and DNA sequence verification, the MBP-(GAGAGS)_n_-GFP recombinant protein was produced in *E. coli* and purified using Ni–NTA affinity chromatography. The recombinant protein was then cut with TEV protease to remove the MBP. The resulting (GAGAGS)_n_-GFP proteins were then purified again using Ni–NTA affinity chromatography and anion exchange chromatography. The nomenclature used is listed in Table 1. The size and purity of the (GAGAGS)_n_-GFP recombinant protein were confirmed by SDS-PAGE analysis (Fig. 2).

**Table 1:** Nomenclature for the conjugates in this work and their molecular weights.

SFMP-fusion protein	Nomenclature	Molecular weight (kDa)
GFP	0R	28.5
(GAGAGS)_1_-GFP	1R	29.2
(GAGAGS)_2_-GFP	2R	29.6
(GAGAGS)_3_-GFP	3R	30.0
(GAGAGS)_4_-GFP	4R	30.4
(GAGAGS)_5_-GFP	5R	30.8
(GAGAGS)_6_-GFP	6R	31.2

### Hydrogel fabrication and gelation-time study

**Figure 3:**
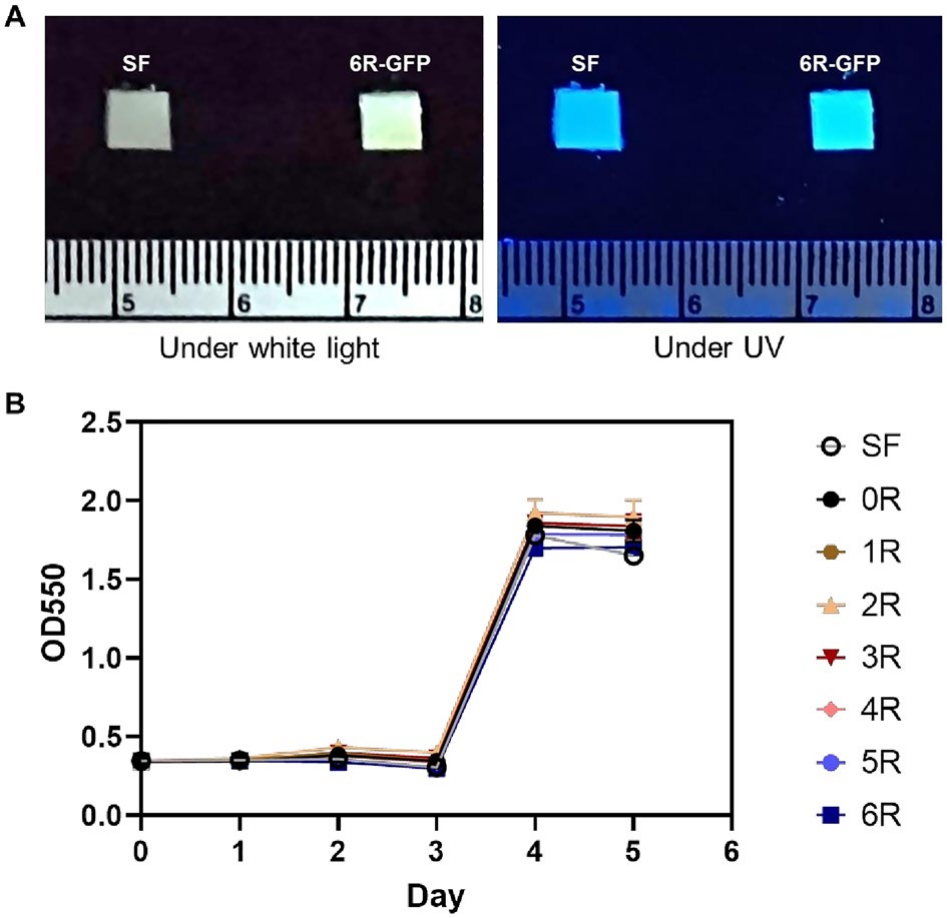
Hydrogel morphology and gelation. (**A**) SF hydrogel (SF) and SF hydrogel containing (GAGAGS)_6_-GFP (6R-GFP) under white light (left), or UV light (right). The scale was in centimeters. (**B**) Gelation time of different hydrogels. The experiment was done in triplicate. The error bars are standard deviations.

The (GAGAGS)_n_-GFP was added to the SF solution prior to its sol–gel transition. We observed no remaining solution after the gelation was completed. This means that the fusion protein at the used concentration was completely incorporated into the hydrogel, and thus the loading efficiency of (GAGAGS)_n_-GFP was expected to be 100%. The green fluorescence could be seen with bare eyes when the hydrogel was exposed to UV light (Fig. 3A). We also examined whether the SFMPs affect the gelation time of the hydrogel. The result showed that all hydrogels had a gelation time of approximately 3.5 days (Fig. 3B). Thus, the SFMPs did not significantly change the gelation time.

### In vitro release of the (GAGAGS)_n_-GFP from the SF hydrogel

**Figure 4:**
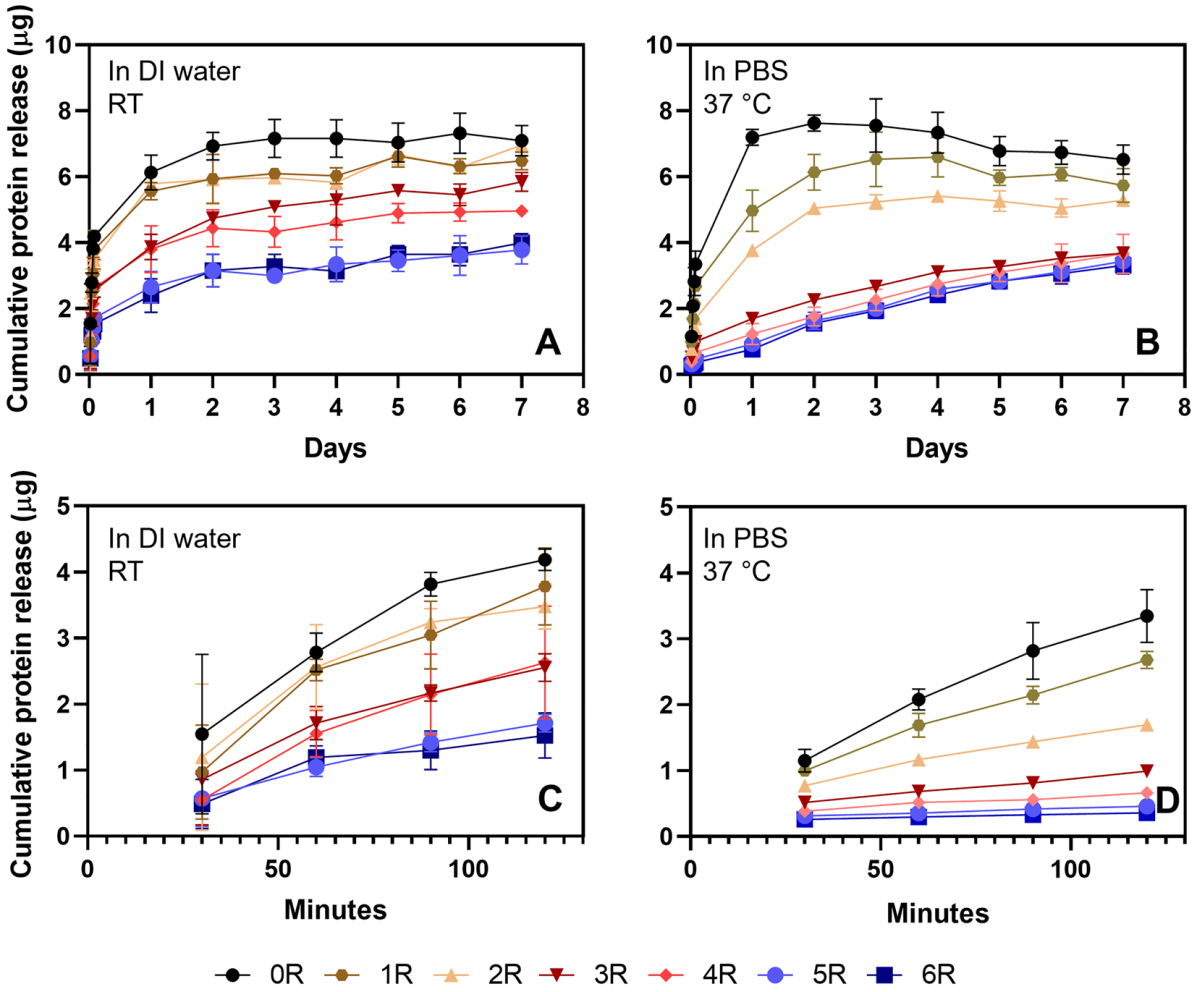
In vitro release profiles of the recombinant GFP from the SF hydrogels in different conditions. The release was studied in water at room temperature (left) and in PBS at 37 °C (right). (**A**,**B**) The release profile of the recombinant GFP in 7 days. (**C**,**D**) The release profiles during the first 2 h, magnified from (**A**) and (**B**), respectively. The experiment was done in triplicate. The error bars are standard deviations.

To analyze the (GAGAGS)_n_-GFP release profiles, the GFP fluorescence intensity of the DI water or PBS in which the hydrogel had been submerged was monitored overtime. In general, the release of all samples could be divided into 2 phases: the quick initial release and the slower release leading to an equilibrium (Fig. 4A and B). A more detailed inspection of the first 2 h revealed that the initial release of the unconjugated-GFP control (0R; black line) was much faster than the (GAGAGS)_n_-GFP (Fig. 4C and D), and the time at which the control reached the equilibrium was earlier than the (GAGAGS)_n_-GFP (Fig. 4A and B). The control and the (GAGAGS)_n=1–2_-GFP took 2 days to reach its equilibrium while the (GAGAGS)_n=3–6_-GFP did not reach their equilibria within 7 days. It is worth noting that the (GAGAGS)_n=3–6_-GFP curves were still increasing. This suggests that the SFMPs could be useful for long-term delivery of tagged molecules from a SF hydrogel platform. When comparing the release in DI water at room temperature and in PBS at 37 °C, the release in the former condition was faster. This indicates that our delivery system responded differently to solvents. Nevertheless, the release rates were inversely proportional to the length of the SFMP tag.

We then further analyzed the initial release during the first 2 h using linear regression (Table 2). The goodness of fit determined from the coefficient of determination (*R*^2^) was close to or equal to 1. As mentioned earlier, we found that there was a negative correlation between the number of SFMP repeats and the release rate shown by the slope. In fact, the unconjugated GFP was released at the rate of 0.029 µg/min in DI water at room temperature and 0.024 µg/min in PBS at 37 °C. The release rate decreased with the SFMP length. The longest SFMP, (GAGAGS)_6_-GFP, was released at the rate of 0.010 µg/min in DI water at room temperature and 0.001 µg/min in PBS at 37 °C.

**Table 2:** Linear regression of the release during the first 2 h.

Conjugates released from SF hydrogel	Release in DI water at 25 °C		Release in PBS at 37 °C	
	*R*^2^	Rate (slope) $\times {10}^{-2}$ µg/min	*R*^2^	Rate (slope) $\times {10}^{-2}$ µg/min
GFP	0.95	2.99	0.99	2.44
GAGAGS-GFP	0.94	2.99	0.99	1.84
(GAGAGS)_2_-GFP	0.90	2.51	0.99	1.02
(GAGAGS)_3_-GFP	0.96	1.85	1.00	0.52
(GAGAGS)_4_-GFP	0.97	2.27	0.96	0.29
(GAGAGS)_5_-GFP	0.99	1.27	0.99	0.17
(GAGAGS)_6_-GFP	0.86	1.01	1.00	0.11

We also noticed that the release profiles of all samples might be divided into 3 groups based on the overlaps of the curves. The grouping was most apparent in DI water (Fig. 4A and B). Group 1 was the GAGAGS-GFP and (GAGAGS)_2_-GFP whose curves overlapped at many time points. Group 2 was the (GAGAGS)_3_-GFP and (GAGAGS)_4_-GFP, with their curves most similar during the initial release. Group 3 was the (GAGAGS)_5_-GFP and (GAGAGS)_6_-GFP that showed overlapping curves throughout our observation.

**Figure 5:**
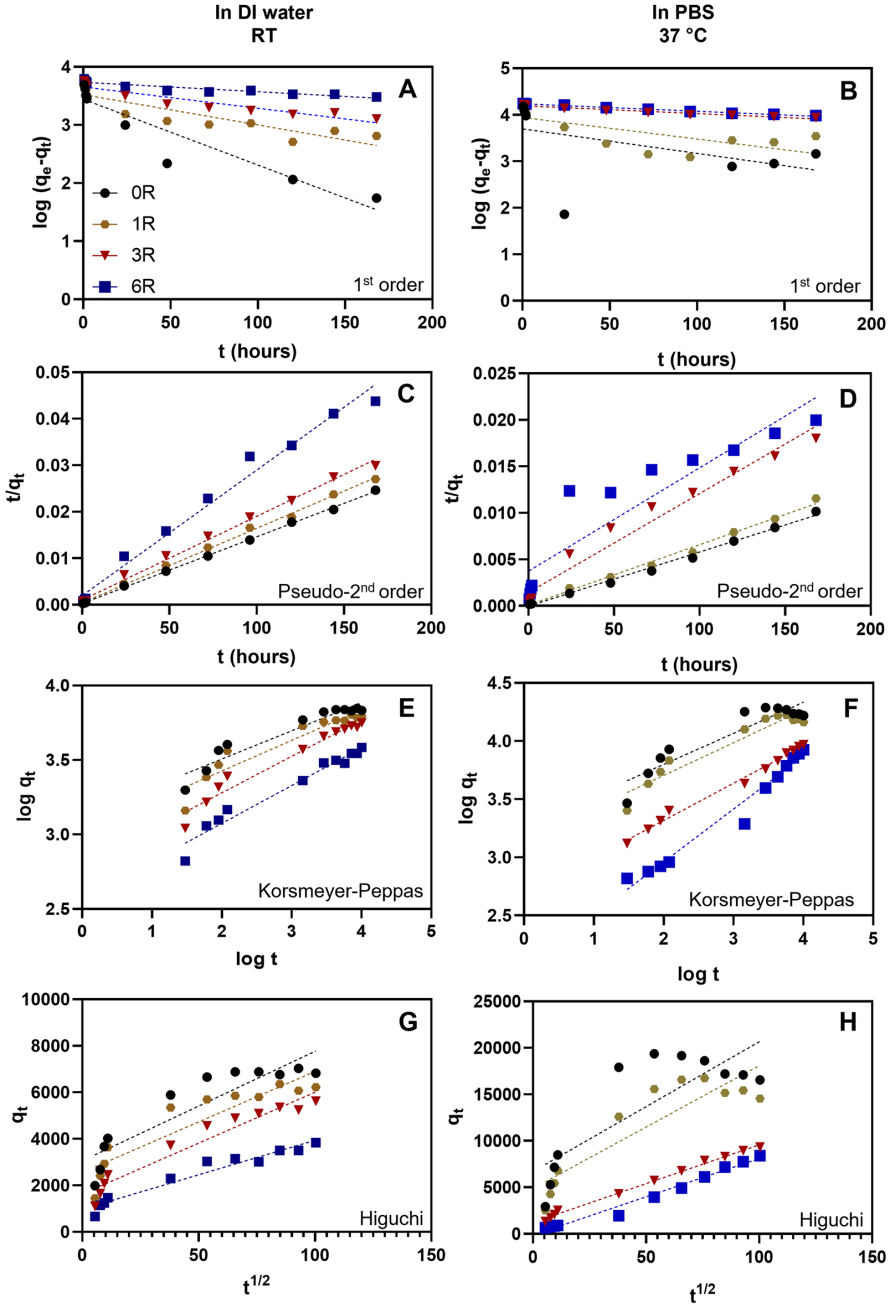
Release kinetics of the recombinant GFP released from the SF hydrogels in different conditions. The release was studied in water at room temperature (left) and in PBS at 37 °C (right). The release profiles were fitted to different kinetic models. (**A**,**B**) First-order model. (**C**,**D**) Pseudo-second-order model. (**E**,**F**) Korsmeyer-Peppas model. (**G**,**H**) Higuchi model.

Furthermore, we attempted to explain the release phenomenon using mathematical release-kinetic models. The above release profiles were fitted to 4 release-kinetic models: first order, pseudo-second order, Korsmeyer-Peppas, and Higuchi model (Fig. 5). The R^2^ and the rate constant (*k*) of each model were shown in Tables 3 and 4. Mostly, the *R*^2^ of the pseudo-second order model was the greatest and closest to 1, implying that the release profiles were best explained using this model. Interestingly, (GAGAGS)_3_-GFP and (GAGAGS)_6_-GFP in PBS at 37 °C were the exception. They best fit the Higuchi and first order model, respectively. Moreover, we found that the rate constants of all models (*k*_*1*_*, k*_*2*_*, k*_*kp*_, and *k*_*H*_) were inversely proportional to the SFMP length. In addition, the release exponents (*n*) in the Korsmeyer-Peppas model increased with the SFMP length although they all were smaller than 0.45, which is the boundary between Fickian and non-Fickian diffusion^19^.

**Table 3:** Values of constants in the kinetic models used in the analysis (in DI water at room temperature).

Conjugates released from SF hydrogel	1st order		Pseudo-2nd order		Korsmeyer-Peppas			Higuchi	
	${k}_{1}$	${R}^{2}$	${k}_{2}$(× 10^9^)	${R}^{2}$	${k}_{KP}$(× 10^3^)	$n$	${R}^{2}$	${k}_{H}$	${R}^{2}$
GFP	− 0.011	0.883	183.067	0.999	1.340	0.190	0.913	47.044	0.821
GAGAGS-GFP	− 0.005	0.808	89.032	0.998	1.038	0.205	0.889	43.379	0.828
(GAGAGS)_3_-GFP	− 0.004	0.899	30.799	0.999	0.617	0.245	0.958	43.737	0.933
(GAGAGS)_6_-GFP	− 0.002	0.884	7.011	0.985	0.369	0.254	0.960	29.617	0.947

${k}_{1}, {k}_{2}, {k}_{KP}, \; {{\text{and}} \; k}_{H}$ are rate constants in the different models.

${R}^{2}$ is the coefficient of determination.

$n$ is the release exponent in the Korsmeyer-Peppas model.

**Table 4:** Values of constants in the kinetic models used in the analysis (in PBS at 37 °C).

Conjugates released from SF hydrogel	1st order		Pseudo-2nd order		Korsmeyer-Peppas			Higuchi	
	${k}_{1}$	${R}^{2}$	${k}_{2}$ (× 10^9^)	${R}^{2}$	${k}_{KP}$ (× 10^3^)	$n$	${R}^{2}$	${k}_{H}$	${R}^{2}$
GFP	− 0.005	0.220	11,101	0.994	1.841	0.267	0.861	139.5	0.675
GAGAGS-GFP	− 0.005	0.508	2,878.9	0.994	1.387	0.281	0.917	132.2	0.793
(GAGAGS)_3_-GFP	− 0.002	0.963	61.814	0.969	0.474	0.320	0.991	83.44	0.995
(GAGAGS)_6_-GFP	− 0.002	0.992	21.728	0.849	0.113	0.454	0.971	82.52	0.981

${k}_{1}, {k}_{2}, {k}_{KP}, \; {{\text{and}} \; k}_{H}$ are rate constants in the different models.

${R}^{2}$ is the coefficient of determination.

$n$ is the release exponent in the Korsmeyer-Peppas model.

### Mechanical analysis and rheology of the SF hydrogel containing (GAGAGS)_n_-GFP

**Figure 6:**
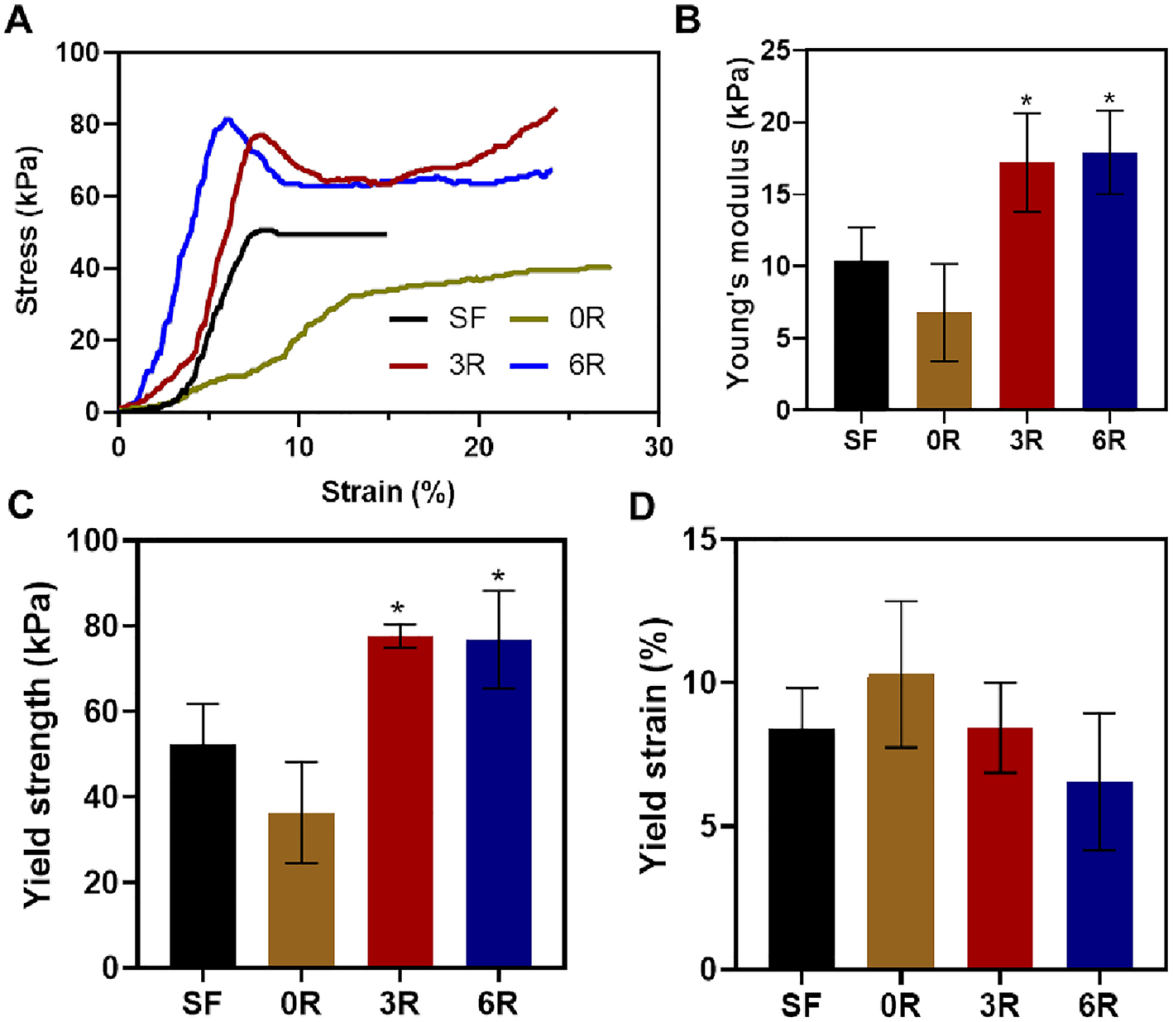
Mechanical properties of hydrogels. (**A**) Representative stress–strain profiles of the hydrogels. (**B**) Young’s modulus. (**C**) Yield strength. (**D**) Yield strain. The experiment was done in triplicate. Each bar represents mean ± SD. *p < 0.05 versus unconjugated GFP (0R).

To further understand how the SFMP-conjugated GFP affects the properties of the SF hydrogel, we conducted mechanical and rheological studies. We chose to test the effect of the (GAGAGS)_3_-GFP and (GAGAGS)_6_-GFP because their sustained-release behaviors were very different from the control, and they could represent the groups mentioned earlier. Uniaxial mechanical analysis of the hydrogels using an unconfined compressive mode revealed mechanical improvement by the SFMP-conjugated GFP. Their representative stress–strain curves are shown in Fig. 6A. The SF hydrogel without SFMP nor GFP had the Young’s modulus and the yield strength of 10.40 ± 2.31 kPa and 52.28 ± 9.50 kPa, respectively (Fig. 6B and C). Addition of the unconjugated GFP reduced those values to 6.78 ± 3.37 kPa and 36.31 ± 11.81 kPa, respectively. The SFMP-conjugated GFP, on the contrary, increased both modulus and strength of the hydrogels to magnitudes significantly greater than those of the control hydrogel. When comparing (GAGAGS)_3_-GFP to (GAGAGS)_6_-GFP, their mechanical properties were similar. We also examined the yield strains and found that the unconjugated GFP led to a slight increase in the yield strain whereas the SFMP-conjugated GFP seemed not to increase the yield strain compared to the control hydrogel (Fig. 6D). The mechanical analysis suggests that the SFMP-conjugated GFP strengthens and stiffens the SF hydrogel.

**Figure 7:**
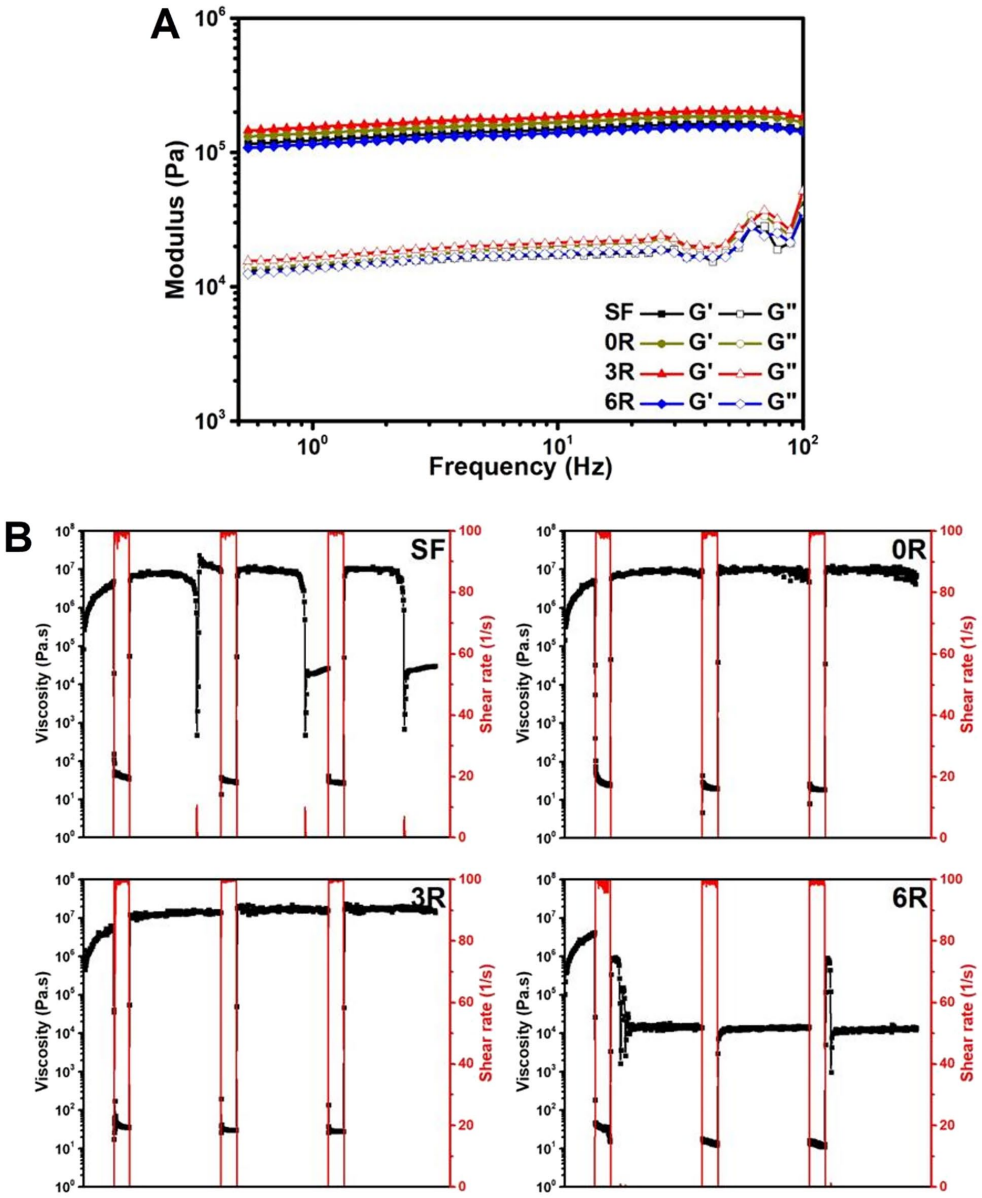
Rheological analysis of hydrogels. (**A**) Viscoelastic property. The storage (G′) and loss (G″) moduli. (**B**) Thixotropic analysis of the hydrogels exposed to an alternate low–high shear-rate regime. The experiment was done in triplicate.

We then studied the viscoelastic properties of the hydrogels using a frequency sweep experiment. All hydrogels had storage moduli (G') greater than loss moduli (G''), suggesting the gel-like behaviours of the hydrogels (Fig. 7A). There was no significant difference between the samples. Furthermore, we assessed the thixotropic properties of the SF hydrogel containing SFMP-conjugated GFP using an alternate high (100 s^−1^) and low (0.1 s^−1^) shear rate for three cycles (Fig. 7B). For the SF hydrogel without SFMP or GFP, the mechanical stability was diminished after applying a high shear rate during 2nd and 3rd cycles. Interestingly, the mechanical strength of the hydrogels with unconjugated GFP or (GAGAGS)_3_-GFP was maintained for the entire 3 cycles of the alternate shearing. The results showed that the hydrogels were viscous (~ 10^7^ Pa·s) when exposed to the low shear rate. They became more fluid (~ 10^1^–10^2^ Pa·s) once the high shear rate was applied, and they could regain the original viscosity when the shear rate returned low. This indicates the shear-thinning properties of the hydrogels. The hydrogel with (GAGAGS)_6_-GFP, however, lost its mechanical stability at the first cycle, as the viscosity was lost and could not be retrieved after the high shear rate. The thixotropic analysis was consistent with the mentioned compressive mechanical analysis, and both point out that an addition of GFP or (GAGAGS)_3_-GFP can enhance the ductility of the hydrogels whereas the (GAGAGS)_6_-GFP can compromise the elasticity of the hydrogels.

### Structural analysis of the SF hydrogel containing (GAGAGS)_n_-GFP

**Figure 8:**
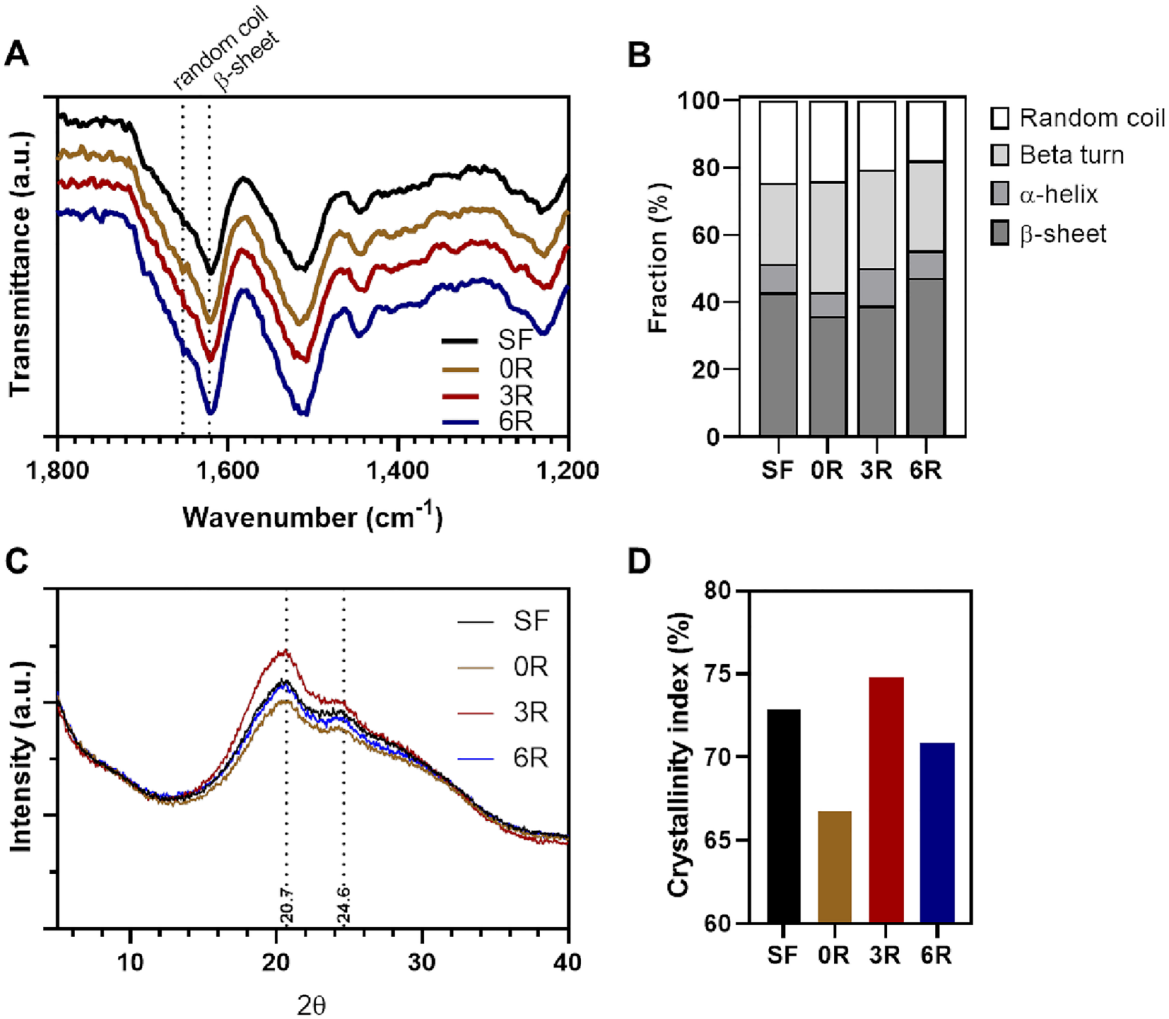
Protein secondary structures and crystallinity study. (**A**) Representative FTIR spectra of the hydrogels. The dotted line labelled random coil is at x = 1653 cm^−1^, and the one labelled β-sheet is at x = 1622 cm^−1^. (**B**) Fractions of the secondary structures in the hydrogels resulted from deconvolution and curve fitting of the FTIR spectra. The FTIR experiment was repeated twice. (**C**) Representative XRD spectra of the hydrogels. The 2θ of 20.7 and 24.6 (dotted lines) are assigned to the crystalline β-sheets. (**D**) The crystallinity indices calculated from the ratio between the area under the curve of the β-sheet crystalline region to the total diffraction area. The XRD experiment was repeated twice.

To examine whether the SFMPs influenced the SF secondary structures after gelation, we performed FTIR and XRD analyses. The FTIR spectra revealed peak shifting, which indicated protein structural changes (Fig. 8A). In the amide I (C=O stretching) region, the sharp dip at 1653 cm^−1^, indicative of random coils, was most obvious in the hydrogel with the unconjugated GFP. The dip became smaller and shifted to 1622 cm^−1^ assigned to β-sheets when SFMP-conjugated GFP had been used (Fig. 8A)^8^. We further quantified the SF secondary structures from the area percentage under defined peaks of Fourier self-deconvoluted FTIR spectra^17^. The results showed that addition of the unconjugated GFP to the hydrogel reduced the crystalline β-sheet structures in the SF hydrogel from 42.76 to 35.87% (Fig. 8B). The (GAGAGS)_3_-GFP recovered the β-sheet fraction back to 39.02%, closer to the control. The (GAGAGS)_6_-GFP increased this fraction to 47.20%, which was clearly greater than that of the control (42.76%). The α-helix fraction showed a similar trend. This fraction was also reduced by the unconjugated GFP and recovered when the SFMP-conjugated GFP was used. Taking these 2 fractions together, it can be concluded that GFP addition compromised the crystalline fraction whereas the SFMPs ameliorated the crystalline structure formation.

In XRD analysis, we found the broad diffraction peaks at 2θ degrees of 20.7 and 24.6 in all hydrogels (Fig. 8C). These peaks represent the β-sheet crystalline structure of SF^18^. When the unconjugated GFP was added to the hydrogel, the intensities of both peaks were smaller compared to the SF hydrogel control. Recovery of the intensities were observed in the hydrogels containing SFMP-conjugated GFP. Furthermore, we quantified the crystallinity index from the ratio between the area under the curve of the β-sheet crystalline region to the total diffraction area^20^. The SF hydrogel control, the hydrogel with unconjugated GFP, the hydrogel with (GAGAGS)_3_-GFP, and the hydrogel with (GAGAGS)_6_-GFP had indices of 72.84, 66.78, 74.79, and 70.83%, respectively (Fig. 8D). The XRD analysis agreed with the FTIR analysis that GFP addition lowered the crystalline fraction whereas the SFMPs helped recover the crystalline fraction.

### Biocompatibility of the SF hydrogel containing (GAGAGS)_n_-GFP

**Figure 9:**
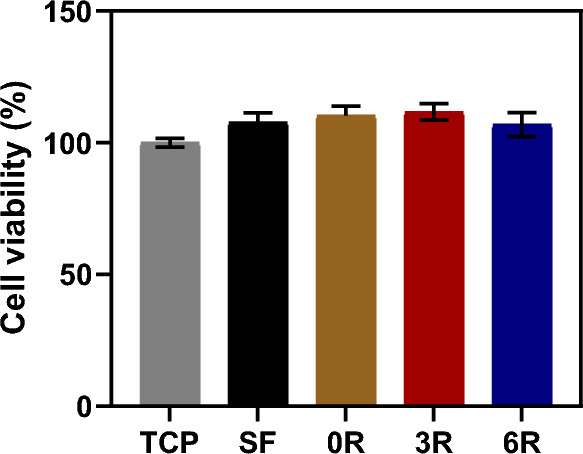
Biocompatibility of hydrogel. Cell viability after the hydrogels were exposed to unloaded SF hydrogel (SF) or SF hydrogel containing unconjugated GFP (0R), (GAGAGS)_3_-GFP (3R), or (GAGAGS)_6_-GFP (6R). Tissue culture plate was used as a control (TCP). The experiment was done in triplicate. Each bar represents mean ± SD.

One of many applications of our SFMP is the controlled release of bioactive molecules for tissue engineering. We thus determined whether the hydrogels were biocompatible. By using MTT assay on the NIH(3T3) fibroblasts after being cultured for 2 days with the hydrogel, we found that the cell viability was not significantly different between different types of hydrogels, and it was not different from the control either (Fig. 9). This was similar to our published work on the biocompatibility of SF-based materials^3,4,21^. The biocompatibility result in this work reassures the safety of silk fibroin for the use at biointerfaces.

## Discussion

Various strategies have been used for controlled release of payloads from SF hydrogel^22,23^. We demonstrated the potential of the biomimetic (GAGAGS)_n_ in optimizing the delivery rate of protein from SF hydrogel. In this work, we used GFP as a model protein to prove our design concept because the release kinetics could be studied at ease. First, we conjugated the SFMPs to GFP by genetic engineering and found the SFMPs did not have a negative effect on the protein, as the GFP fluorescence could still be seen regardless of the SFMP length used in the study. Thus, it is possible that SFMP might be compatible with other proteins as well, but this will likely need to be tested on a case-by-case basis. When the SFMP-GFP was incorporated into the SF solution, it did not interfere with the sol–gel transition because the gelation time remained the same as the control.

Next, we sought to test whether the SFMPs could prolong the GFP release as hypothesized. The in vitro release study in DI water or PBS revealed that the unconjugated GFP loaded into the SF hydrogel burst-released within 2 h. This could be because the unconjugated GFP interfered with the crystalline β-sheets formation. As a result, there were less crystalline parts acting as diffusion barrier. This explanation was supported by the FTIR and XRD results that less crystalline β-sheets were present. As a result, the GFP readily diffused out of the hydrogel when submerged in the DI water or PBS. In contrast, the (GAGAGS)_n_-GFP was able to interact with the β-sheets. The β-sheets, therefore, trapped the (GAGAGS)_n_-GFP in the crystalline regions. The FTIR and XRD analyses reaffirmed this argument, as more crystalline β-sheets were seen when (GAGAGS)_n_-GFP had been used. We also believed that the hydrophobic β-sheets acted as a diffusion barrier for the soluble (GAGAGS)_n_-GFP. As a result, the GFP release was prolonged by the SFMPs in a length-dependent manner. In addition to that, we found that the release in PBS at 37 °C was slower than that in DI wat at room temperature. The slower release could be explained by less swelling in PBS due to electrostatic shielding demonstrated in previous work^24,25^. A slight decrease in cumulative protein release in PBS at 37 °C compared to water at room temperature was observed after the third day of the experiment. We reason that this could be due to degradation of GFP at higher temperature over a long period of time.

To support the argument that the SFMP helped prolong the release by affixing the pendant GFP to the crystalline β-sheets, the SF structural transition between amorphous and crystalline structures was examined using FTIR and XRD. By comparing to the unconjugated GFP, the SFMPs reduced the amorphous structures and increased the crystalline structures. This could be concluded that the SFMPs induced a structural transition from amorphous structures to crystalline β-sheets in the SF hydrogel. The increase in the fraction of crystalline β-sheets could be explained by two possibilities. First, the SFMPs were physically incorporated into the β-sheet structures. Alternatively, the SFMPs interacted amongst themselves to form crystalline units that self-aggregated, eventually mixing in the SF hydrogel. We speculated that the latter was unlikely because the molecular weight of our SFMPs could be too small to self-aggregate. There were several reports showing that peptides mimicking different primary structures of the spider silk protein could not aggregate to form nanoparticles or fibrils when their molecular weight was smaller than 5.8 kDa^26,27^. In fact, the molecular weight of our SFMPs ranged only from 0.4 to 2.8 kDa. Therefore, we asserted that the SFMPs helped affix the GFP into the β-sheets in the SF hydrogel rather than self-aggregated within the hydrogel matrix.

The non-linear curve fitting to the kinetic models suggests that our system can be best described mostly by the second-order model, as this model had the R^2^ closest to 1. Furthermore, the rate constants in all mathematical models were inversely proportional to the SFMP length, meaning that the release rate was slower when the SFMP was longer. We also noticed that the release exponents (*n*) in the Korsmeyer-Peppas model increased with the SFMP length although they all were smaller than 0.45, which is the boundary between Fickian and non-Fickian diffusion. Taken these together, the non-linear regression suggests that the GFP was released in all systems predominantly by diffusion, and the diffusion was slowed down when the SFMP length was greater.

Controlling protein release from SF by adjusting the crystallinity was shown earlier with a strategy different from this work. In those work, the percentage of crystalline β-sheets was adjusted by NaCl-treatment time or water-annealing time and the release of horseradish peroxidase (HRP), interferon gamma (IFN-ɣ), or interleukin-4 (IL-4) was studied^28,29^. They showed that the longer treatment time, which led to more β-sheets, resulted in a slower release in the initial burst-release phase. A similar conclusion can be drawn from our work. The β-sheets seem to play a role in limiting the protein release. However, we believed that the main factor that controlled the GFP release in our work was the molecular interaction between the SFMP and the β-sheets in SF rather than the crystallinity. This is because the (GAGAGS)_3_-GFP and (GAGAGS)_6_-GFP led to hydrogels with similar β-sheet fractions, but their release profiles were significantly different. In fact, the initial release rate for the (GAGAGS)_6_-GFP was almost twice as slow as that of the (GAGAGS)_3_-GFP. To conclude, the interaction with the β-sheets rather than the amount of the β-sheets is the main rate-determining factor in our strategy.

To induce the crystalline β-sheet formation in SF, chemical and physical factors can be used such as surfactants, water-annealing, alcohols, mechanical shears, and irradiation^30–35^ Nevertheless, less is known about the β-sheet induction by peptides and proteins. We showed in this work that the addition of SFMPs could promote the β-sheets formation. Interestingly, the GFP protein, which has 11 β-sheets arranged into a β barrel^36^, disrupted the β-sheets formation in SF.

In addition to the release, we characterized the mechanical and rheological properties of the resulting hydrogels. It is established that crystalline β-sheets predominantly determine the mechanical strength while amorphous regions provide elasticity^37,38^. We found that the hydrogels with SFMP-GFP had the compressive strength and stiffness greater than the one with the unconjugated GFP. This could also be explained by the greater percentage of the β-sheets present in the SFMP-GFP-containing hydrogels.

Sustained protein release from silk fibroin hydrogel can be used in diverse applications. Tissue engineering is one of those that requires continuous supply of bioactive protein such as growth factors at a low concentration. The prolonged release of the bioactive protein would ensure tissue regeneration without lacking important signal during the process. Because the SFMP-GFP release profiles were slower in both DI water and PBS compared to the unconjugated GFP, we anticipated that the SFMP-GFP would be released slower than the unconjugated GFP in other systems as well, not limited to applications in tissue engineering. In addition to tissue engineering, our system may be useful for construction materials^39^ where sustained release of enzymes upon material breakage is preferable. Other potential application is the sustained release of protein-based biostimulants for long-term plant growth enhancement^40^.

The rheological study revealed the viscoelastic behaviors of the hydrogels. All of them had a storage modulus (G′) greater than a loss modulus (G″). This means that the hydrogels behaved as an elastic solid^41^. In the thixotropic analysis, the hydrogel under a high/low shear-rate regime showed different responses. The SF hydrogel and the hydrogel with (GAGAGS)_6_-GFP lost their stability after exposure to a high shear rate. In contrast, the hydrogel with the unconjugated GFP and (GAGAGS)_3_-GFP well-recovered after high-to-low shear transition. This demonstrated the shear-thinning properties of the hydrogels. The differences in ductility of the hydrogels indicated by the yield strains could be responsible for the different thixotropic responses. The thixotropic behavior is a desirable characteristic for materials to be used in pharmaceutical and cosmetic preparations^42^.

## Conclusions

The biomimetic (GAGAGS)_n_ does not alter the function of the conjugated protein while prolonging the protein release. The (GAGAGS)_n_-conjugated protein is affixed into the SF hydrogel, mainly by interacting with the crystalline β-sheets. This interaction leads to the sustained release of the conjugated protein. Selecting a proper length of the SFMPs will allow us to optimize the release rate and the hydrogel stability to suit various applications. In addition, using genetically engineered bacteria to produce the (GAGAGS)_n_-conjugated protein will allow scale-up manufacturing.

### Supplementary Information

Supplementary Information.

## Data Availability

The data and materials used in this research are available from the corresponding authors upon a reasonable request.
