# Supplementary material for: Optimizing protein delivery rate from silk fibroin hydrogel using silk fibroin-mimetic peptides conjugation
Source: Sci Rep. 2024 Feb 23;14:4428. doi: 10.1038/s41598-024-53689-7 (PMC10891107; doi:10.1038/s41598-024-53689-7)
Supplement: Supplementary file 1 — Supplementary Information. [file 41598_2024_53689_MOESM1_ESM.docx]

**Optimizing protein delivery rate from silk fibroin hydrogel using silk fibroin-mimetic peptides conjugation**

Jaturong Promsuk^a,b^, Juthatip Manissorn^c,*^, Chavee Laomeephol^c,d^, Jittima Amie Luckanagul^c,d^, Apipon Methachittipan^e^, Khaow Tonsomboon^f^, Ratchapol Jenjob^g^, Sugeun Yang^g^, Peerapat Thongnuek^c,h,i*^, Kittikhun Wangkanont^a,b*^

^a^ Center of Excellence for Molecular Biology and Genomics of Shrimp, Department of Biochemistry, Faculty of Science, Chulalongkorn University, Bangkok 10330 Thailand

^b^ Center of Excellence in Molecular Crop, Department of Biochemistry, Department of Biochemistry, Faculty of Science, Chulalongkorn University, Bangkok 10330 Thailand

^c^ Biomedical Materials and Devices for Revolutionary Integrative Systems Engineering Research Unit (BMD-RISE), Faculty of Engineering, Chulalongkorn University, Bangkok 10330, Thailand

^d^ Department of Pharmaceutics and Industrial Pharmacy, Faculty of Pharmaceutical Sciences, Chulalongkorn University, Bangkok 10330, Thailand

^e^ Nano Engineering Program, International School of Engineering, Faculty of Engineering, Chulalongkorn University, Bangkok 10330 Thailand

^f^ National Center for Genetic Engineering and Biotechnology (BIOTEC), National Science and Technology Development Agency (NSTDA), Khlong Luang, Pathum Thani 12120, Thailand

^g^ Department of Biomedical Science, BK21 FOUR program in Biomedical Science and Engineering, Inha University College of Medicine, Incheon 22332, Republic of Korea

^h^ Biomedical Engineering Program, Faculty of Engineering, Chulalongkorn University, Bangkok 10330 Thailand.

^i^ Biomedical Engineering Research Center, Faculty of Engineering, Chulalongkorn University, Bangkok 10330 Thailand.

^*^ Corresponding authors

**Corresponding Authors**

Juthatip Manissorn (m_juthatip@yahoo.com), Peerapat Thongnuek (peerapat.t@chula.ac.th), Kittikhun Wangkanont (kittikhun.w@chula.ac.th)

**Table S1**. Nucleotide sequences of primers used for construction of (GAGAGS)_n_-GFP expression plasmids. The restriction sites are underlined.

| Primer | Nucleotide sequence |
| --- | --- |
| Primer A | 5ʹ-TATACCATGGGCGGTGCTGGTGCTGGCTCAGGTGCTGGTGCTGGATCTGGAGCAGGAGCAGGAAGT-3ʹ |
| Primer B | 5ʹ- CTGGATCTGGAGCAGGAGCAGGAAGTGGTGCTGGCTCTGGTTCATCAGGTATGGTGAGCAAGGGCGAGGAG-3ʹ |
| Primer C | 5ʹ-AGTGCGGCCGCCTTGTACAGCTCGTCCATGCCGAG-3ʹ |
| Primer D | 5ʹ-TGTCCATGGGCGAAAACCTGTATTTTCAGGGTGGTGCTGGTGCTGGCTCAGGTGCTG-3ʹ |
| Primer E | 5ʹ-TGTCCATGGGCGAAAACCTGTATTTTCAGGGTGCTGGCTCTGGTTCATCAGGTAT-3ʹ |
| Primer F | 5ʹ-CGCGGATCCTTATCAGTGGTGGTGGTGGTGGTGCTCG-3ʹ |
| Primer G | 5ʹ-ATGTCCATGGGCGAAAACCTGTATTTTCAGGGTGGTGCTGGTGCTG  GCTCAGGTGCTGGCTCTGGTTCATCAGGTATGGTG-3ʹ |
| Primer H | 5ʹ-ATGTCCATGGGCGAAAACCTGTATTTTCAGGGTGGTGCTGGTGCTG  GCTCAGGTGCTGGTGCTGGCTCA -3ʹ |
| Primer I | 5ʹ-GGGGCCGGCGCTGGCTCGGGTGCTGGTGCTGGCTCA-3ʹ |
| Primer J | 5ʹ-ATGTCCATGGGCGAAAACCTGTATTTTCAGGGTGGAGCTGGCGCA  GGTTCTGGGGCCGGCGCTGGCTCG-3ʹ |
| Primer K | 5ʹ-ATGTCCATGGGCGAAAACCTGTATTTTCAGGGTGGTGCTGGTGCTGGCTCTGGAGCAGGAGCAGGATCTGGGGCCGGCGCTGGCTCG -3ʹ |

**Table S2**. Amino acid sequences of (GAGAGS)_n_-GFP proteins. “/” = TEV cleavage site.

| Protein name | Amino acid sequence |
| --- | --- |
| MBP-TEV-GFP | MBP-ISHMSMGENLYFQ/GAGSGSSGMVSKGEELFTGVVPILVE  LDGDVNGHKFSVSGEGEGDATYGKLTLKFICTTGKLPVPWPTLVTTLTYGVQCFSRYPDHMKQHDFFKSAMPEGYVQERTIFFKDDGNYKTRAEVKFEGDTLVNRIELKGIDFKEDGNILGHKLEYNYNSHNVYIMADKQKNGIKVNFKIRHNIEDGSVQLADHYQQNTPIGDGPVLLPDNHYLSTQSALSKDPNEKRDHMVLLEFVTAAGITLGMDELYKAAALE*HHHHHH* |
| MBP-TEV-GAGAGS-GFP | MBP-ISHMSMGENLYFQ/GGAGAGSGAGSGSSGMVSKGEELFTG  VVPILVELDGDVNGHKFSVSGEGEGDATYGKLTLKFICTTGKLPVPWPTLVTTLTYGVQCFSRYPDHMKQHDFFKSAMPEGYVQERTIFFKDDGNYKTRAEVKFEGDTLVNRIELKGIDFKEDGNILGHKLEYNYNSHNVYIMADKQKNGIKVNFKIRHNIEDGSVQLADHYQQNTPIGDGPVLLPDNHYLSTQSALSKDPNEKRDHMVLLEFVTAAGITLGMDELYKAAALE*HHHHHH* |
| MBP-TEV-(GAGAGS)_2_-GFP | MBP-ISHMSMGENLYFQ/GGAGAGSGAGAGSGAGSGSSGMVSK  GEELFTGVVPILVELDGDVNGHKFSVSGEGEGDATYGKLTLKFICTTGKLPVPWPTLVTTLTYGVQCFSRYPDHMKQHDFFKSAMPEGYVQERTIFFKDDGNYKTRAEVKFEGDTLVNRIELKGIDFKEDGNILGHKLEYNYNSHNVYIMADKQKNGIKVNFKIRHNIEDGSVQLADHYQQNTPIGDGPVLLPDNHYLSTQSALSKDPNEKRDHMVLLEFVTAAGITLGMDELYKAAALE*HHHHHH* |
| MBP-TEV-(GAGAGS)_3_-GFP | MBP-ISHMSMGENLYFQ/GGAGAGSGAGAGSGAGAGSGAGSGSS  GMVSKGEELFTGVVPILVELDGDVNGHKFSVSGEGEGDATYGKLTLKFICTTGKLPVPWPTLVTTLTYGVQCFSRYPDHMKQHDFFKSAMPEGYVQERTIFFKDDGNYKTRAEVKFEGDTLVNRIELKGIDFKEDGNILGHKLEYNYNSHNVYIMADKQKNGIKVNFKIRHNIEDGSVQLADHYQQNTPIGDGPVLLPDNHYLSTQSALSKDPNEKRDHMVLLEFVTAAGITLGMDELYKAAALE*HHHHHH* |
| MBP-TEV-(GAGAGS)_4_-GFP | MBP-ISHMSMGENLYFQ/GGAGAGSGAGAGSGAGAGSGAGAGS  GAGSGSSGMVSKGEELFTGVVPILVELDGDVNGHKFSVSGEGEGDATYGKLTLKFICTTGKLPVPWPTLVTTLTYGVQCFSRYPDHMKQHDFFKSAMPEGYVQERTIFFKDDGNYKTRAEVKFEGDTLVNRIELKGIDFKEDGNILGHKLEYNYNSHNVYIMADKQKNGIKVNFKIRHNIEDGSVQLADHYQQNTPIGDGPVLLPDNHYLSTQSALSKDPNEKRDHMVLLEFVTAAGITLGMDELYKAAALE*HHHHHH* |
| MBP-TEV-(GAGAGS)_5_-GFP | MBP-ISHMSMGENLYFQ/GGAGAGSGAGAGSGAGAGSGAGAGS  GAGAGSGAGSGSSGMVSKGEELFTGVVPILVELDGDVNGHKFSVSGEGEGDATYGKLTLKFICTTGKLPVPWPTLVTTLTYGVQCFSRYPDHMKQHDFFKSAMPEGYVQERTIFFKDDGNYKTRAEVKFEGDTLVNRIELKGIDFKEDGNILGHKLEYNYNSHNVYIMADKQKNGIKVNFKIRHNIEDGSVQLADHYQQNTPIGDGPVLLPDNHYLSTQSALSKDPNEKRDHMVLLEFVTAAGITLGMDELYKAAALE*HHHHHH* |
| MBP-TEV-(GAGAGS)_6_-GFP | MBP-ISHMSMGENLYFQ/GGAGAGSGAGAGSGAGAGSGAGAGS  GAGAGSGAGAGSGAGSGSSGMVSKGEELFTGVVPILVELDGDVNGHKFSVSGEGEGDATYGKLTLKFICTTGKLPVPWPTLVTTLTYGVQCFSRYPDHMKQHDFFKSAMPEGYVQERTIFFKDDGNYKTRAEVKFEGDTLVNRIELKGIDFKEDGNILGHKLEYNYNSHNVYIMADKQKNGIKVNFKIRHNIEDGSVQLADHYQQNTPIGDGPVLLPDNHYLSTQSALSKDPNEKRDHMVLLEFVTAAGITLGMDELYKAAALE*HHHHHH* |


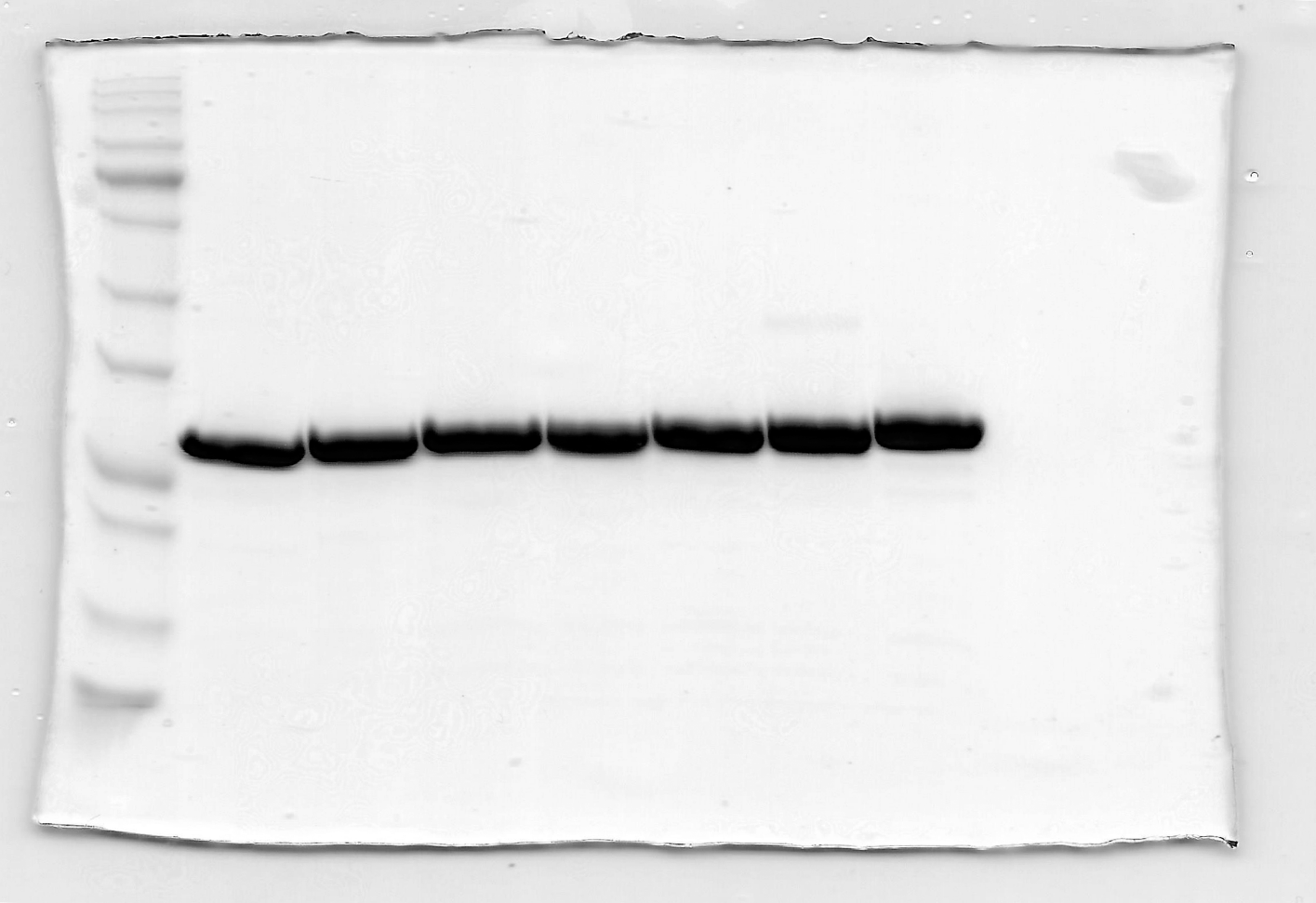


**Figure S1**. Full SDS-PAGE gel of Figure 2.
